# Biochemical Properties of a New Cold-Active Mono- and Diacylglycerol Lipase from Marine Member *Janibacter* sp. Strain HTCC2649

**DOI:** 10.3390/ijms150610554

**Published:** 2014-06-12

**Authors:** Dongjuan Yuan, Dongming Lan, Ruipu Xin, Bo Yang, Yonghua Wang

**Affiliations:** 1 College of Light Industry and Food Sciences, South China University of Technology, Guangzhou 510640, China; E-Mails: dongjuanyuan@gmail.com (D.Y.); dmlan@scut.edu.cn (D.L.); catherinexin0116@sina.com (R.X.); 2 School of Bioscience and Bioengineering, South China University of Technology, Guangzhou 510006, China; E-Mail: yangbo@scut.edu.cn

**Keywords:** *Janibacter* sp., mono- and di-acylglycerol lipase, biochemical characterization, camellia oil

## Abstract

Mono- and di-acylglycerol lipase has been applied to industrial usage in oil modification for its special substrate selectivity. Until now, the reported mono- and di-acylglycerol lipases from microorganism are limited, and there is no report on the mono- and di-acylglycerol lipase from bacteria. A predicted lipase (named MAJ1) from marine *Janibacter* sp. strain HTCC2649 was purified and biochemical characterized. MAJ1 was clustered in the family I.7 of esterase/lipase. The optimum activity of the purified MAJ1 occurred at pH 7.0 and 30 °C. The enzyme retained 50% of the optimum activity at 5 °C, indicating that MAJ1 is a cold-active lipase. The enzyme activity was stable in the presence of various metal ions, and inhibited in EDTA. MAJ1 was resistant to detergents. MAJ1 preferentially hydrolyzed mono- and di-acylglycerols, but did not show activity to triacylglycerols of camellia oil substrates. Further, MAJ1 is low homologous to that of the reported fungal diacylglycerol lipases, including *Malassezia globosa* lipase 1 (SMG1), *Penicillium camembertii* lipase U-150 (PCL), and *Aspergillus oryzae* lipase (AOL). Thus, we identified a novel cold-active bacterial lipase with a *sn*-1/3 preference towards mono- and di-acylglycerides for the first time. Moreover, it has the potential, in oil modification, for special substrate selectivity.

## 1. Introduction

Lipases (E.C. 3.1.1.3) are ubiquitous enzymes that catalyze hydrolysis and synthesis of acylglycerol and other water insoluble esters [1], and they have been used in many industries such as the food, leather, detergent and paper industries [2]. Different lipases show different substrate specificity. Most lipases exhibit the activity to triacylglycerol (TAG), diacylglycerol (DAG), and monoacylglycerol (MAG). Only a few of them from fungi have recently been found to be strictly specific for mono- and di-acylglycerol, such as *Malassezia globosa* lipase1 (SMG1) [3], *Penicillium camembertii* lipase U-150 [4], and *Aspergillus oryzae* lipase (AOL) [5]. There is no report on mono- and di-acylglycerol lipase from bacteria strain. Mono- and di-acylglycerol lipase has been applied to industrial usage in oil modification for its special substrate selectivity. For example, lipase SMG1 is used to synthesize DAG product with high concentration [6,7]. It has also been proved that mono- and di-acylglycerol lipase from *Penicillium camembertii* and monoacylglycerol lipase from *Bacillus* sp. Strain H-257 can be used to obtain DAG with high purity [8]. Lipase SMG1 could not hydrolyze soybean oil, however, it can enhance the hydrolysis of soybean oil by combining with Palatase 20000L (an *sn*-1,3 specific triacylglycerol lipase) [6].

Marine environment covers more than half of Earth’s surface and represents the most unexplored habitat. It has the great potential offering abundant resources for research and development. Up to now, a large number of lipases from terrestrial organisms have been reported, but the lipases from marine microorganisms are severely limited. There are some reports on the triacylglycerol lipases from marine microorganism strains. EML1 from the deep-sea sediment metagenome is a cold-active lipase and displays more than 50% activity at 5 °C [9]. The M37 lipase from *Photobacterium lipolyticum* sp. nov. displays a maximum activity at 25 °C and maintains its activity at a low temperature range from 5 to 25 °C [10]. The purified lipase from marine strain *Pseudomonas otitidis* is stable at pH 5.0–9.0 and temperature 30–80 °C [11]. However, there is also no report on the mono- and diacylglycerol lipase from marine bacteria strain.

Until now, the sequences of 4479 microorganisms in National Center for Biotechnology Information (NCBI) microbial genome data from prokaryotic genome sequencing projects were determined. The abundant annotated lipases provide the possibility to explore the novel enzymes with specific properties for industry usage [2]. Furthermore, the promotion in the classification of lipase in different families with functionally relevant residues will also aid in the selection of the novel lipases [12]. In this study, nine annotated lipase genes were found in the genome sequence of marine *Janibacter* sp. strain HTCC2649. *Janibacter* sp. strain HTCC2649 is a novel marine member of the *Actinobacteria*, family *Intrasporangiaceae*, and is closely related to *Janibacter melonis* CM2104T and *Knoellia sinensis* HKI 0119T [13]. This organism has the unique phylogenetic position among the phylum of *Actinobacteria*. Through the combination of sequence alignment and cluster analysis and lipase classification, a possible novel lipase was selected and biochemically characterized.

## 2. Results and Discussion

## 2.1. Sequence Analysis and Identification of MAJ1

There are nine predicted lipases in the draft genome sequence of marine *Janibacter* sp. strain HTCC2649. To see how these lipases were related to known esterases/lipases, the phylogenetic relationship was analyzed based on the esterase/lipase classification (family I–VIII) [2,14]. Nine predicted lipases were clustered into different families (Figure S1). Only one gene of marine *Janibacter* was clustered in the family I (named MAJ1), and the deduced amino acid sequence of MAJ1 encoding a putative alpha/beta hydrolase. Four genes classified into the group of family IV and V, respectively. Others were difficult to classify by the phylogenetic analysis because of the low sequence similarity. 

**Figure 1:**
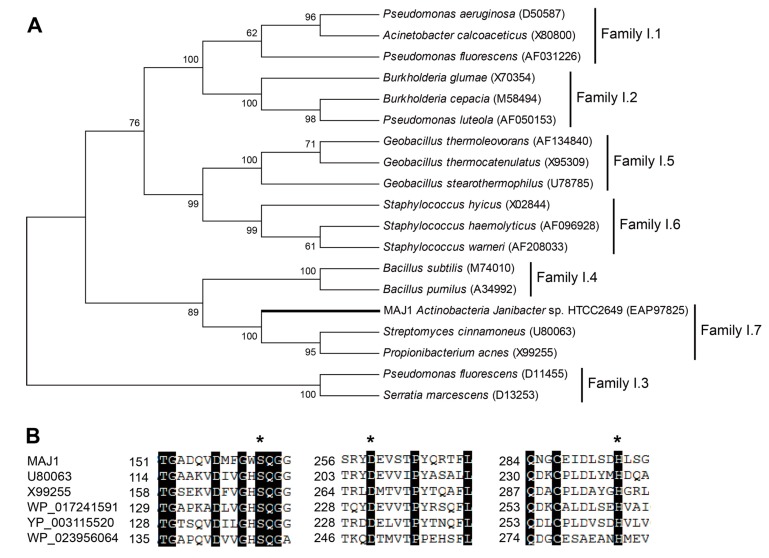
Gene features of MAJ. (**A**) Phylogenetic tree of the lipase family I. The tree was constructed using the MEGA 6.0 program with the neighbor-joining algorithm. Bar: 0.2 substitutions per amino acid site; and (**B**) Multiple sequence alignment of MAJ1 and other related proteins. U80063: lipase from *Streptomyces cinnamoneus*; X99255: lipase from *Propionibacterium acnes*; WP_017241591: lipase 2 from *Streptomyces* sp.; YP_003115520: lipase 2 from *Catenulispora acidiphila*; WP_023956064: lipase from *Williamsia* sp. D3. The asterisk indicates three catalytic residues of serine, aspartate, and histidine residue.

The MAJ1 was found to be 987 bp long, with an Open Reading Frame (ORF) encoding 328 aa long protein. The deduced protein was a typically secreted lipase, containing a 32 amino acids signal peptide and a lipase 2 domain. MAJ1 was grouped into the lipase subfamily I.7 (Figure 1A), sharing 40% amino acid identity with the putative lipase *Streptomyces cinnamoneus* (U80063). MAJ1 contains the typical active site of the α/β hydrolase fold enzyme. The three catalytic residues (serine, aspartate, and histidine) of MAJ1 form the typical catalytic triad by the multiple alignment analysis (Figure 1B), and the nucleophilic serine residue locates at a highly conserved G-X-S-Q-G pentapeptide motif (Figure 1B). MAJ1 also contains the oxyanion hole region of PVVLVHG as the typical catalytic center that is located at 79–85, but HG sequence for the oxyanion hole was not observed in other two putative lipases (U80063 and X99255) in subfamily I.7. Subfamily I.7 belongs to the group of lipase class 2 that are not clearly related to other lipase families. Biochemical characters of family I.7 are also not clear, and no structure of the member in this family has yet been solved. From the sequence alignment and cluster analysis, it seems likely that MAJ1 could be a novel molecular entity unrelated to the known lipases.

## 2.2. Expression, Purification, and Characterization of MAJ1

**Figure 2:**
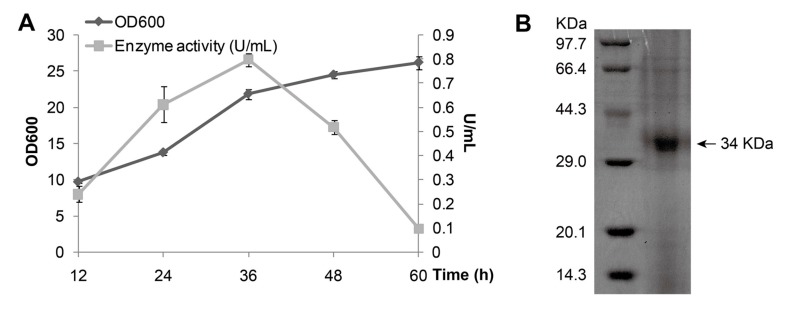
Expression and purification of MAJ1. (**A**) Growth curve of *P. pastoris* X-33 with MAJ1; and (**B**) Purification of MAJ1. The enzyme activity refers to the activity of the supernatant of fermentation broth.

MAJ1 gene fused with *C*-terminal His-tag was cloned in a *Pichia pastoris* (*P. pastoris*) constitutive expression vector pGAPZαA with α-factor secretion signal and then expressed in *P. pastoris* X-33 strain (Figure 2A). The growth curve indicated that the maximum production of MAJ1 (0.8 U/mL) was obtained at 36 h when the cell density at 600 nm reached around 25. The secreted protein in supernatant was purified by metal-chelating using Ni-NTA column, and then analyzed by 15% Sodium Dodecyl Sulphate Polyacrylamide Gel Electrophoresis (SDS-PAGE). SDS-PAGE analysis of purified MAJ1 showed a single band corresponding to about 34 kDa which correlated well to the predicted length of MAJ1 (Figure 2B).

## 2.3. Effects of Temperature on Lipase Activity and Stability

**Figure 3:**
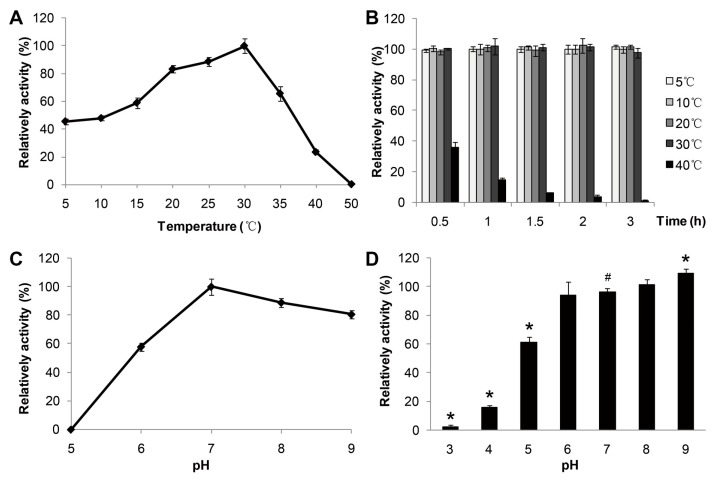
Effects of temperature and pH on the enzyme activity of MAJ1. (**A**) The enzyme activity was measured at various temperatures at pH 7.0. The value obtained at 30 °C was taken as 100%; (**B**) The enzyme activity was determined at 30 °C by using *p*-nitrophenyl caprate (C10) after treated with 2 h at different temperatures. The value obtained at 30 °C was taken as 100%; (**C**) The enzyme activity was measured at various pH. The value obtained at pH 7.0 was taken as 100%; and (**D**) The enzyme activity was determined at 30 °C by using *p*-nitrophenyl caprate (C10) after treated with 12 h at various pH. The value obtained at pH 7.0 was taken as 100%. # compared to the value at pH 7.0, *p* < 0.05; * compared to the value at pH 7.0, *p* < 0.01.

The effect of temperature (Figure 3A,B) on the lipase activities and the stability of MAJ1 were determined spectrophotometrically using *p*-nitrophenol caprate (C10) as substrate. The purified enzyme showed an optimal activity at 30 °C (Figure 3A) and displayed about 50% of the optimal activity at 5 °C. Furthermore, the enzyme activity declined to lower than 20% of the optimal activity above 40 °C, indicating good stability of the lipase activity of MAJ1 at low temperature. It has been reported that a lipase from deep-sea area of Edison Seamount showed an optimal temperature at 25 °C and displayed more than 50% activity at 5 °C, and the lipase was identified to be cold-active enzyme [9]. Thus, MAJ1 could be classified as cold-active enzyme based on the temperature profiles. The thermo-stability of recombinant MAJ1 was investigated by incubation at different temperature for 3 h at pH 7.0. The relative activity of recombinant MAJ1 was greater than 95% after incubation of MAJ1 at temperature of 5, 10, 20 and 30 °C for 3 h, while its activity decreased dramatically after the incubation temperature of 40 °C (Figure 3B).

## 2.4. Effects of pH on Lipase Activity and Stability

The effect of pH on the activity and the stability of MAJ1 were investigated. The results were shown in Figure 3C,D. The recombinant MAJ1 was found to be most active at a neural or slightly alkaline pH (pH 7.0–8.0) and exhibited very low activity (<10%) at pH 5.0 (Figure 3C). Thus, the configuration of enzyme might be destroyed at low pH value, which resulted in decreasing the activity of enzyme. The similar results were observed with lipases from *Candida albicans*, *Pseudomonas mandelii* and *Bacillus coagulans* BTS-3 [15,16,17]. The enzyme stability was tested after incubation at different pH value buffer for 12 h. The activity of MAJ1 decreased by 40% after incubation at buffer with the pH 5.0 for 12 h, while it was relatively stable in an alkaline environment (Figure 3D).

## 2.5. Effect of Chemicals, Detergents and Water-Miscible Solvents

To evaluate the effect of various chemical agents, detergents, and water-miscible solvents on the lipolytic activity of MAJ1, purified protein was incubated with various metal ions and chemical agents, and the remaining activity was measured with *p*-nitrophenyl caprate (C10) as substrate at 30 °C. The activity of MAJ1 was stable in the presence of Zn^2+^, Cu^2+^, Mg^2+^, Ca^2+^, Ni^2+^, Mn^2+^, K^+^ and Na^+^, and was completely inhibited by EDTA (Table 1). The addition of 1 mM EDTA significantly decreased the enzyme activity, and various metal ions could not affect the enzyme activity. Further, the changes of enzyme activity with the increase of Na^+^ concentration (from 1 to 4 mM) were not significant (Table 1).

Different surfactants have different influences on MAJ1 activity. For anionic surfactant, the enzyme activity of MAJ1 decreased after being treated with di-octyl sulfosuccinate (Aerosol-OT, AOT) and sodium dodecyl sulfate (SDS), it still retained 79.12% of maximal activity when MAJ1 was treated with *N*-lauroyl sarcosine sodium for 2 h. AOT and SDS placed an inhibitory effect on activity of MAJ1 may be its negative charge and its denaturing capability. For cationic surfactant, tetradecyl trimethyl ammonium bromide (TTAB), cetyl trimethyl ammonium bromide (CTAB), octadecyl trimethyl ammonium bromide (OTAB) significantly decreased the enzyme activity. The result suggests that the positive charge may also dramatically inhibit the enzyme activity. For zwitterionic surfactant, the stability of enzyme activity decreased in the presence of soy lecithin, but this effect was not observed in 3-tetradecyl dimethyl sulfopropyl betaine. In nonionic surfactant, relative activities of MAJ1 were 30.72%, 13.25% and 4.98% when 1% of Triton X-100 (HLB-13.0), Tween-80 (HLB-15.0), and Tween-20 (HLB-16.7) were used (Table 2). The results suggest that MAJ1 activities decreased with the increment of hydrophilic-lipophilic balance (HLB) value. The results indicated that the hydrophilicity of non-ionic surfactants might decrease MAJ1 stability. Though MAJ1 activity decreased after being treated with methanol and ethanol, it still retained more than 62% of maximal activity when MAJ1 was incubated with acetone and isopropanol for 2 h. It was reported that the hydrophilic solvents may strip off the water layer from the surface of the enzyme and compete strongly for hydrogen bonds between protein atoms, leading to protein unfolding and subsequent denaturation [18].

**Table 1:** Effect of various metal ions on the enzyme activity of MAJ1.

Metal Ions (1 mM) or EDTA (1 mM)	Relative Activity (%)
None	100.00 ± 0.00
ZnSO_4_	91.34 ± 3.20
CuSO_4_	92.13 ± 5.21
MgSO_4_	93.13 ± 2.67
CaCl_2_	95.72 ± 1.97
NiCl_2_	91.21 ± 3.00
MnSO_4_	95.96 ± 3.01
MnCl_2_	94.01 ± 6.45
K_2_SO_4_	93.89 ± 6.40
MgCl_2_	93.79 ± 2.01
EDTA	32.29 ± 2.90
NaCl (1 mM)	90.41 ± 2.60
NaCl (2 mM)	95.54 ± 3.71
NaCl (3 mM)	97.12 ± 2.04
NaCl (4 mM)	97.76 ± 3.67
NaCl (5 mM)	95.27 ± 4.86

**Table 2:** Effect of various detergents and organic solvents on the enzyme activity of MAJ1.

	Detergent (1%)	Relative Activity (%)
	None	100.00 ± 0.00
Anionic surfactant	di-Octyl sulfosuccinate (Aerosol-OT, AOT)	15.76 ± 2.02
	*N*-Lauroyl sarcosine sodium	79.12 ± 1.04
	Sodium dodecyl sulfate (SDS)	13.88 ± 1.41
Cationic surfactant	Tetradecyl trimethyl ammonium bromide (TTAB)	21.20 ± 2.05
	Cetyl trimethyl ammonium bromide (CTAB)	43.76 ± 3.17
	Octadecyl trimethyl ammonium bromide (OCAB)	28.92 ± 1.45
Zwitterionic surfactant	3-Tetradecyl dimethyl sulfopropyl betaine	21.24 ± 3.39
	Soy lecithin	82.84 ± 2.74
Nonionic surfactant	Tween-20	4.98 ± 0.73
	Tween-60	13.27 ± 0.30
	Tween-80	13.25 ± 0.62
	Triton X-100	30.72 ± 1.47
	Nonylphenol ethoxylates	45.74 ± 3.98
	Polyethylene oxide lauryl ether (Brij-35)	84.57 ± 2.82
Organic solvents	Methanol	18.54 ± 4.43
	Alcohol	32.64 ± 4.38
	Isopropanol	62.51 ± 4.58
	Acetone	64.18 ± 3.59

## 2.6. Substrate Specificity of MAJ1

**Figure 4:**
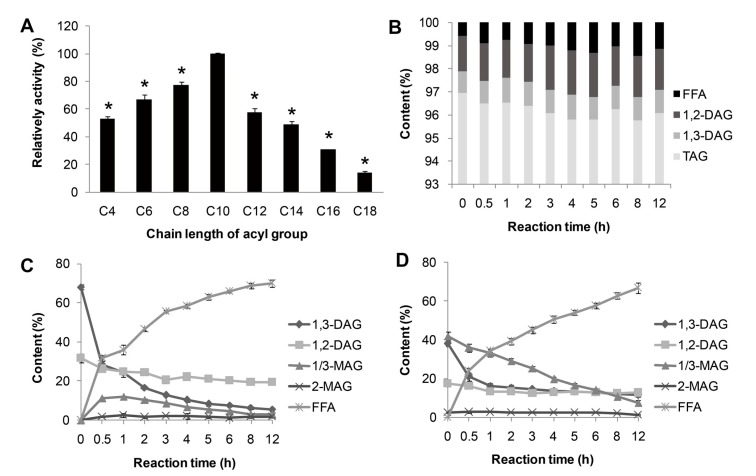
Substrate specificity of MAJ1. (**A**) Substrate specificity of MAJ1 toward various *p*-nitrophenyl esters; * compared to the relative activity of C10, *p* < 0.01; (**B**) Chemical composition (%) of fractions during hydrolysis of triacylglycerol (TAG)-enriched camellia oil; (**C**) Chemical composition (%) of fractions during hydrolysis of diacylglycerol (DAG)-enriched oil; and (**D**) Chemical composition (%) of fractions during hydrolysis of DAG: monoacylglycerol (MAG) (1:1)-enriched oil.

The substrate specificity of lipase MAJ1 was investigated. The results of the reactions by the artificial substrate of *p*-nitrophenyl esters, and natural substrates of TAG, DAG and MAG of camellia oil were shown in Figure 4. MAJ1 could hydrolyze different *p*-nitrophenyl esters with different chain lengths (C4 to C18) and showed the highest preference toward *p*-nitrophenyl caprate (C10) (Figure 4A). For natural substrate, lipase MAJ1 showed specific activity to DAG and MAG (Figure 4C,D), but no activity to TAG (Figure 4B and Table S1). The ratio of 1,3-DAG to 1,2-DAG decreased sharply from 2.12 to 0.28 and from 2.20 to 0.91 with the reaction proceeding in the DAG substrate and DAG: MAG (1:1) mixture substrates, respectively (Tables S2 and S3). The results suggest that the enzyme is 1,3-specific lipase. It has been proved that mono- and di-acylglycerol lipase could be used to obtain TAG with high purity [19]. Thus, lipase MAJ1 would have potential applications in industries for oil modification and production of partial glycerides.

In this study, we characterized the first mono- and di-acylglycerol lipase from bacteria that was stable at low temperature from marine *Janibacter* sp. strain HTCC2649. This result suggests that the temperature property of lipolytic enzyme from marine might be reflected by their environmental characters. It was also confirmed in other reports from the environment with low temperature to develop the cold-active enzymes. The cold-active lipase from an Antarctic deep sea psychrotrophic bacterium *Pseudomonas* sp. 7323 has the optimal enzyme activity at temperature of 30 °C [20]. The cold-adapted lipase from *Photobacterium lipolyticum* sp. nov. isolated from an intertidal flat of the Yellow Sea displays a maximum activity at 25 °C and maintains its activity at a low temperature range from 5 to 25 °C [10].

## 3. Experimental Section

## 3.1. Bacterial Strains, Chemicals, Plasmids and Medium

*Escherichia coli* DH5α (Stratagene, LaJolla, CA, USA) was used as host strain for cloning and the plasmid pGAPZαA (Invitrogen, Carlsbad, CA, USA) was used as cloning vector. *P. pastoris* X-33 (Invitrogen) strain was used for expression. The *p*-nitrophenol and *p*-nitrophenyl esters were purchased from Sigma-Aldrich. Camellia oil was purchased from the local shop in China. *n*-Hexane and 2-propanol of HPLC grade were purchased from Kermel Chemical Reagent (Tianjin, China). All other chemicals were of analytical grade.

## 3.2. Sequence Data and Phylogenetic Study

The lipase protein sequences were obtained from the UniProt protein sequence database or the NCBI GenBank. Phylogenetic analysis was based on protein sequences, and the alignment was obtained with the CLUSTALX V2 program. Phylogenetic analysis with Neighbor-joining (NJ) was conducted using MEGA 6.0 software [21].

## 3.3. Gene and Expression Plasmids

The MAJ1 gene (UniProtKB accession number A3TMR7, from *Actinobacteria Janibacter* sp. strain HTCC2649) without signal peptide was artificially synthesized by Sangon Biotech (Shanghai, China) according to the code usage of *P. pastoris*. The code-optimized MAJ1 gene was cloned into pGAPZαA (Invitrogen) vector and was controlled by the promoter of the glyceraldehyde-3-phosphate dehydrogenase (GAPDH). The sequence was confirmed by DNA sequencing and then pGAPZαA-MAJ1 plasmid linearized by restriction enzyme *Bln* I and transformed into *P. pastoris* X-33 strain by electroporation. The transformants were selected on YPD (1% (*w*/*v*) yeast extract, 2% (*w*/*v*) peptone and 2% (*w*/*v*) glucose) plates supplemented with Zeocin (100 µg/mL) at 30 °C for three days.

## 3.4. Expression and Purification of MAJI

The *P. pastoris* X-33 cells containing the recombinant plasmids were grown and expressed in YPD liquid medium at 30 °C with shaking of 200 rpm for 36 h. The supernatant of fermentation broth after centrifugation (10,000× *g*, 20 min, 4 °C) was filtered through a 0.22 μm filter membrane by suction filtration, then concentrated and buffer-exchanged to buffer A (20 mM sodium phosphate, 0.5 M NaCl, 30 mM imidazole, pH 7.4, at 4 °C) through a 10 kDa molecular mass membrane (Vivaflow 200, Sartorius, Germany). The MAJ1 was purified by metal-chelating chromatography using HisTrap HP column (GE Healthcare, Buckinghamshire, UK). The buffer A containing crude enzyme was loaded into a chromatographic column and washed with buffer B (20 mM sodium phosphate, 0.5 M NaCl, 500 mM imidazole, pH 7.4). The purified lipases liquid was collected and analyzed by 15% SDS-PAGE. Protein concentrations were determined by the BCA Protein Assay Kit (Sangon Biotech Shanghai Co., Ltd., Shanghai, China).

## 3.5. Biochemical Properties of MAJI

The lipase activity of MAJ1 was assayed with purified lipase obtained following metal-chelating chromatography. The enzyme activity was determined by colorimetric techniques, using *p*-nitrophenyl esters as the substrate [22]. The reaction mixture consisted of 80 μL buffer of desired pH value, 10 μL enzyme liquid of appropriate concentration, 10 μL 10 mM substrate dissolved in ethanol. The reaction was incubated at desired temperature for 5 min, and terminated by adding 100 μL 1% SDS. The absorbance of the reaction mixture was measured at 405 nm. One unit of enzyme activity is defined as the amount of enzyme required to release 1 μmol of *p*-nitrophenol per minute. All the results in this study were the mean of triplicate measurements.

Substrates specificity for MAJ1 was studied using 10 mM *p*-nitrophenyl ester dissolved in ethanol with different chain length: *p*-nitrophenyl butyrate (C4), *p*-Nitrophenyl caproate (C6), *p*-nitrophenyl caprylate (C8), *p*-nitrophenyl caprate (C10), *p*-nitrophenyl laurate (C12), *p*-nitrophenyl myristate (C14), *p*-nitrophenyl palmitate (C16).

Optimum pH for the purified lipase was determined using pH buffer solutions ranging from 3.0 to 8.0 (pH 3: 0.2 M Na_2_HPO_4_-0.1 M citric acid, pH 4–5: 0.1 M sodium citrate-0.1 M citric acid, pH 6–7: 0.1 M phosphate buffer, pH 8: 0.05 M Tris-HCl) at 30 °C. The pH stability of these lipases were determined by pre-incubating those enzymes in different pH buffers (from pH 3–9) for 12 h at 4 °C followed by measuring residual activity at 30 °C in buffer of pH 7.0.

The optimum temperature of the lipase was determined by measuring the activity at temperatures ranging from 5 to 50 °C at pH 7.0. The temperature stability of these lipase were determined by incubating the lipase MAJ1 at 20, 30 and 40 °C for 2 h. Samples were taken for measurement of residual activity under standard assay conditions (pH 7.0, optimum temperature).

Various metal ions (ZnSO_4_, CuSO_4_, MgSO_4_, CaCl_2_, NiCl_2_, MnSO_4_, MnCl_2_, K_2_SO_4_, MgCl_2_, NaCl) and EDTA at final concentrations of 1 mM were added to the enzyme in pH 7.0, then assayed for lipase activity after preincubation at 4 °C for 2 h. Effect of detergents on lipase activity was determined by incubating the enzyme for 2 h at 4 °C in pH 7.0 containing 1% (*w*/*v*) of the different detergents. Lipase activity was measured at the beginning and end of the incubation period. The activity of the enzyme preparation in the absence of detergent before incubation was defined as the 100% level.

## 3.6. Preparation and Purification of DAG and MAG by Molecular Distillation

Free fatty acids (FFAs) were prepared by enzymatic hydrolysis of TAG from camellia oil, and then the products were further purified by molecular distillation. High purity of DAG and MAG were synthesized by enzymatic esterification of glycerol and FFAs from camellia oil with catalysis of *Penicillium camembertii* lipase, and then the products were further purified by molecular distillation [8].

## 3.7. Hydrolysis of TAG, DAG, and MAG of Camellia Oil by MAJ1

Partial hydrolytic reactions were carried out in a 15 mL conical flask with stirring at 200 rpm. The reaction condition of water content (25%, with respect to oil), enzyme load (45 U/g, with respect to oil), temperature (30 °C) were investigated their effects on the degree of hydrolysis of lower glycerides (referring to MAG and DAG). MAJ1 could be oxidized for longer reaction time, and the partial glyceride would not be hydrolyzed completely for shorter reaction time. Therefore, reaction time was fixed to 12 h. Aliquots (150 μL) of the reaction mixture were periodically withdrawn from the reactions and then were centrifuged at 10, 000× *g* for 3 min to remove the water then the upper layer. Twenty μL of supernatants were diluted in 1 mL of *n*-hexane/2-propanol/methanoic acid (15:1:0.003 *v*/*v*/*v*) for HPLC analysis. The experiments were performed in triplicate and the results were presented as the average.

## 3.8. HPLC Analysis of the Products in Hydrolytic Reaction

The content of acylglycerols and free FA in the hydrolytic products was analyzed by a Waters 2695 HPLC with a Waters 2414 parallax refractive index detector on a Phenomenex Luna silica column (Phenomenex Corporation, Torrance, CA, USA, 250 mm × 4.6 mm i.d., 5 μm particle size). The mobile phase was a mixture of *n*-hexane/2-propanol/formic acid (15:1:0.003 *v*/*v*/*v*) with a flow rate of 1.0 mL/min. Peaks in HPLC were identified by comparison of their retention times with reference standards. Peak-areas percentages were calculated using Waters 2695 integration software. Analysis was repeated for triplication, and the results were presented as the average of triplicate measurements of the samples.

## 3.9. Statistical Analysis

All analytical determinations were carried out in triplicate. The results are reported as the means ± standard deviations (SD) of these measurements. Significant differences in the means were accomplished by using an ANOVA procedure (*p* < 0.05).

## 4. Conclusions

In short, we identified a novel cold-active lipase with a *sn*-1/3 preference towards mono- and di-acylglycerols from specific marine microorganism *Janibacter* sp. strain HTCC2649. It represents an example that the genome sequence can provide a chance to develop the novel enzyme with specific biochemical characterization.

## Supplementary Files

Supplementary File 1 Supplementary Information (PDF, 623 KB)
