# Supplementary material for: Biochemical Properties of a New Cold-Active Mono- and Diacylglycerol Lipase from Marine Member Janibacter sp. Strain HTCC2649
Source: Int J Mol Sci. 2014 Jun 12;15(6):10554–66. doi: 10.3390/ijms150610554 (PMC4100168; doi:10.3390/ijms150610554)
Supplement: Supplementary File 1 — Supplementary Information (PDF, 623 KB) [file ijms-15-10554-s001.pdf]

Supplementary Information

Figure S1. Phylogenetic tree of 9 predicted lipases from marine *Janibacter* sp. strain HTCC2649 and putative lipases from different families. The tree was constructed using the MEGA 6.0 program with the neighbor-joining algorithm using bootstrap method and pairwise deletion. Bar: 0.2 substitutions per amino acid site. The red line shows the predicted lipases from marine *Janibacter* sp. strain HTCC2649.

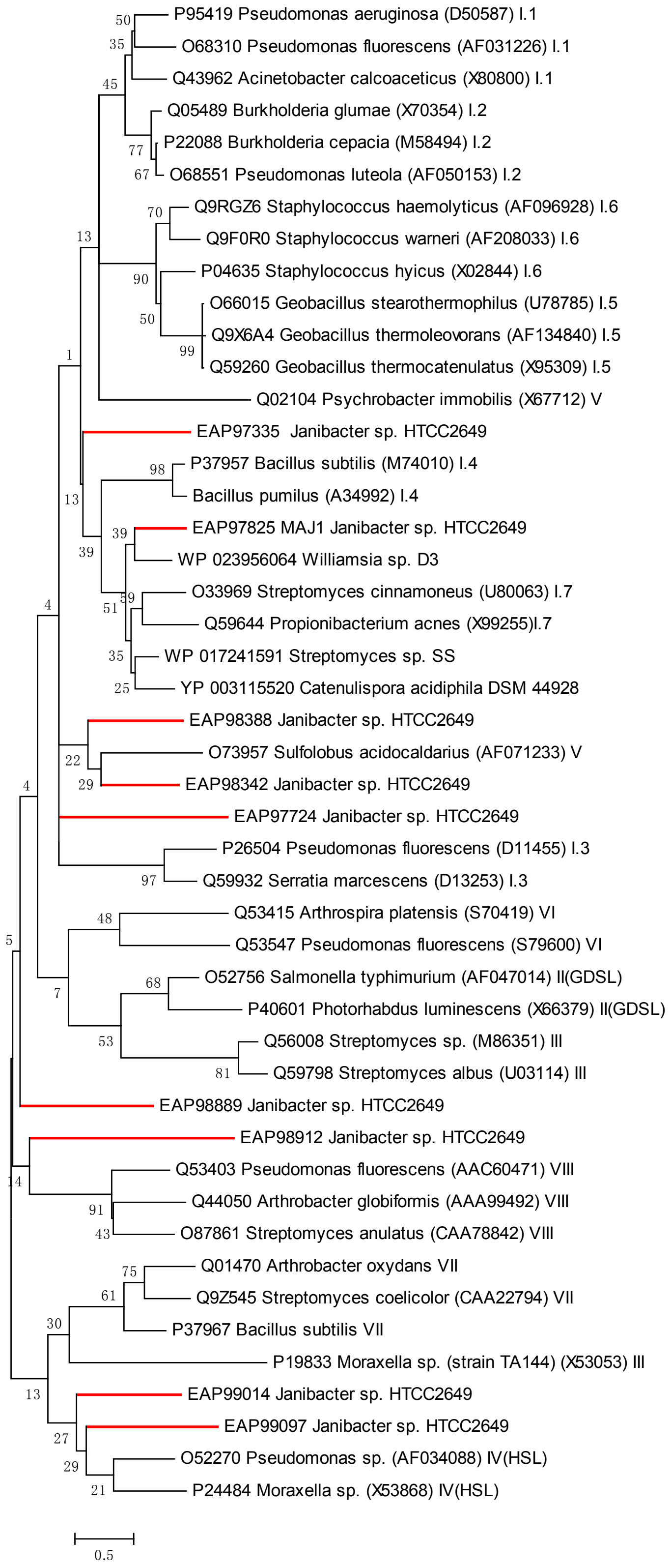

Table S1. Chemical composition (%) of fractions during hydrolysis of TAG-enriched camellia oil.

Hours	TAG	1,3-DAG	1,2-DAG	FFAs
0 h	96.90 ± 0.18	0.95 ± 0.03	1.67 ± 0.15	0.63 ± 0.15
0.5 h	96.49 ± 0.25	0.97 ± 0.09	1.65 ± 0.24	0.89 ± 0.14
1 h	96.52 ± 0.46	1.09 ± 0.09	1.62 ± 0.32	0.77 ± 0.16
2 h	96.41 ± 0.34	1.03 ± 0.04	1.62 ± 0.28	0.94 ± 0.15
3 h	96.10 ± 0.03	1.00 ± 0.11	1.90 ± 0.06	1.00 ± 0.08
4 h	95.81 ± 0.14	1.06 ± 0.07	1.91 ± 0.06	1.21 ± 0.08
5 h	95.80 ± 0.14	0.97 ± 0.07	1.91 ± 0.11	1.31 ± 0.07
6 h	96.26 ± 0.19	1.00 ± 0.23	1.70 ± 0.32	1.04 ± 0.09
8 h	95.77 ± 0.29	0.99 ± 0.15	1.77 ± 0.11	1.47 ± 0.22
12 h	96.09 ± 0.11	1.01 ± 0.15	1.76 ± 0.19	1.14 ± 0.05

Table S2. Chemical composition (%) of fractions during hydrolysis of DAG-enriched camellia oil.

Hours	1,3-DAG	1,2-DAG	1/3-MAG	2-MAG	FFAs
0 h	67.89 ± 2.14	31.95 ± 2.01	0.00 ± 0.00	0.00 ± 0.00	0.00 ± 0.00
0.5 h	28.44 ± 0.74	26.36 ± 0.97	11.30 ± 0.23	1.98 ± 0.25	31.91 ± 1.36
1 h	24.24 ± 2.26	24.86 ± 1.02	12.10 ± 0.14	2.69 ± 0.95	36.10 ± 2.58
2 h	16.75 ± 0.53	24.41 ± 1.22	10.55 ± 0.46	1.67 ± 0.65	46.62 ± 0.96
3 h	12.79 ± 0.25	20.59 ± 1.14	8.63 ± 0.21	2.28 ± 0.24	55.71 ± 0.57
4 h	10.32 ± 0.82	22.28 ± 1.09	6.74 ± 0.27	2.28 ± 1.51	58.38 ± 1.08
5 h	8.25 ± 0.86	21.25 ± 0.98	5.66 ± 0.49	1.89 ± 0.49	62.95 ± 1.57
6 h	7.72 ± 0.66	20.44 ± 0.76	4.62 ± 0.69	1.34 ± 0.58	65.89 ± 0.63
8 h	6.37 ± 0.63	19.64 ± 0.89	3.13 ± 0.44	1.98 ± 0.92	68.88 ± 1.39
12 h	5.53 ± 0.91	19.44 ± 0.87	2.96 ± 0.17	1.97 ± 0.40	70.10 ± 1.94

Table S3. Chemical composition (%) of fractions during hydrolysis of DAG: MAG (1:1)-enriched oil.

Hours	1,3-DAG	1,2-DAG	1/3-MAG	2-MAG	FFA
0 h	38.25 ± 1.39	17.42 ± 1.96	42.33 ± 2.02	2.44 ± 0.29	0.00 ± 0.00
0.5 h	21.09 ± 2.15	16.26 ± 1.33	35.94 ± 2.20	2.81 ± 0.49	23.92 ± 1.65
1 h	16.14 ± 1.43	13.31 ± 1.15	33.41 ± 2.12	2.77 ± 0.17	34.36 ± 0.57
2 h	15.47 ± 1.22	13.47 ± 0.48	29.28 ± 1.12	2.46 ± 0.36	39.31 ± 1.56
3 h	14.41 ± 0.91	12.60 ± 0.56	25.28 ± 1.18	2.59 ± 0.34	45.11 ± 1.65
4 h	13.75 ± 0.97	13.07 ± 0.58	19.76 ± 1.30	2.69 ± 0.19	50.73 ± 1.75
5 h	13.27 ± 0.45	13.34 ± 0.77	16.69 ± 0.90	2.58 ± 0.03	54.12 ± 1.18
6 h	12.89 ± 0.63	13.05 ± 0.54	13.99 ± 1.19	2.44 ± 0.07	57.63 ± 1.40
8 h	11.96 ± 0.28	12.51 ± 0.50	10.66 ± 0.97	2.04 ± 0.09	62.83 ± 1.60
12 h	11.55 ± 0.81	12.73 ± 0.64	7.48 ± 1.53	1.40 ± 0.29	66.84 ± 2.58

© 2014 by the authors; licensee MDPI, Basel, Switzerland. This article is an open access article distributed under the terms and conditions of the Creative Commons Attribution license (http://creativecommons.org/licenses/by/3.0/).
